# Editorial: Understanding and engineering cyber-physical collectives

**DOI:** 10.3389/frobt.2024.1407421

**Published:** 2024-05-06

**Authors:** Roberto Casadei, Lukas Esterle, Rose Gamble, Paul Harvey, Elizabeth F. Wanner

**Affiliations:** ^1^ Department of Computer Science and Engineering, Alma Mater Studiorum—Università di Bologna, Cesena, Italy; ^2^ Department of Electrical and Computer Engineering, Aarhus University, Aarhus, Denmark; ^3^ Tandy School of Computer Science, University of Tulsa, Tulsa, OK, United States; ^4^ Raukuten Mobile Innovation Studio, Tokyo, Japan; ^5^ James Watt School of Engineering, University of Glasgow, Glasgow, United Kingdom; ^6^ School of Engineering and Applied Science, Aston University, Birmingham, United Kingdom

**Keywords:** cyber-physical collectives, swarm intelligence, collective intelligence, multi-robot systems, multi-agent systems, complex adaptive systems, internet of things, large-scale cyber-physical systems

## 1 Introduction

*Cyber-physical collectives (CPCs)* are systems consisting of groups of interactive computational devices situated in physical space. Their emergence is fostered by recent techno-scientific trends like the Internet of Things (IoT), cyber-physical systems (CPSs), pervasive computing, and swarm robotics. Such systems feature networks of devices that are capable of computation and communication with other devices, as well as sensing, actuation, and physical interaction with their environment. This distributed sensing, processing, and action enables them to address spatially situated problems and provide environment-wide services through their *collective intelligence (CI)* in a wide range of domains including smart homes, buildings, factories, cities, forests, oceans, and so on. However, the inherent complexity of such systems in terms of heterogeneity, scale, non-linear interaction, and emergent behaviour calls for scientific and engineering ideas, methods, and tools (cf. Wirsing et al. (2023); Dorigo et al. (2021); Brambilla et al. (2013); Casadei (2023a; b)). This Research Topic gathers contributions related to *understanding and engineering cyber-physical collectives*.

CPCs can be approached from several theoretical and engineering perspectives. Informative, but non-exhaustive, exemplars include:

• *cyber-physical and hybrid systems* (Cassandras and Lafortune, 2008; Kim and Kumar 2012);
• *coordination models and languages* (Malone and Crowston, 1994; Ciatto et al., 2020);
• *autonomic computing* and self-* systems (Kephart and Chess, 2003; de Lemos et al., 2010; Bellman et al., 2021; Harvey et al., 2021);
• *artificial life* (Aguilar et al., 2014; Gershenson, 2023);

• *multi-agent systems (MASs)* (Wooldridge, 2009; Mascardi et al., 2019; Boissier et al., 2020);
• *grammar systems* (Klavins et al., 2006; Csuhaj-Varjú et al., 2018);
• *swarm robotics* (Brambilla et al., 2013; Dorigo et al., 2021);
• *collective intelligence* (Malone and Bernstein, 2022; Casadei, 2023a);
• *complex adaptive systems* (Bucchiarone and Mongiello, 2019; Abeywickrama et al., 2020; Wirsing et al., 2023);
• *multi-agent reinforcement* learning (Zhang et al., 2019); and
• *macro-programming* (Júnior et al., 2022; Casadei, 2023b).

An excellent source of inspiration comes from natural systems, whose study may promote the identification of novel bio-inspired algorithms of collective behaviour (Fernandez-Marquez et al., 2013; Roli and Braccini, 2018). Common classes of collective behaviour include (i) spatially-organising behaviours, (ii) collective movement, and (iii) collective decision making. Their design can be carried out by *automatic* techniques (e.g., leveraging evolutionary computing or multi-agent reinforcement learning—see also Buchberger (2023)) or *manual programming* techniques (e.g., leveraging macro-programming)—or combinations thereof (Aguzzi et al., 2022). Macro-programming can be supported by high-level programming languages and libraries (e.g., Pinciroli and Beltrame, 2016; Aguzzi et al., 2023), possibly founded on formal calculi such as *field calculi* (Viroli et al., 2019) and *dynamic ensemble/attribute-based calculi* (Alrahman et al., 2020) that can promote model-checking, verification of properties, and formal guarantees of implemented behaviours. Such algorithmic and language-based approaches also promote the identification of reusable *patterns* of collective behaviour and organisation (Horling and Lesser, 2004; Pianini et al., 2021). A crucial aspect of macro-systems is also their *efficient deployment* on heterogeneous infrastructures across the *edge-cloud continuum* (Casadei et al., 2022; Júnior et al., 2022). Another key theme is the integration of artificial collective systems with humans, leading to notions like *human-in-the-loop* cyber-physical systems (Annaswamy et al., 2023), *social machines* (Hendler and Berners-Lee, 2010), and *complex socio-cognitive systems* (Galesic et al., 2023). Last but not least, given CPCs are quite peculiar systems due to their complexity, they come with new challenges and opportunities regarding *digital twins* (Casadei et al., 2021), *security and privacy* (Aldini, 2018), and *green computing* (Stolfi and Alba, 2018).

As discussed and summarised in the following, this Research Topic addresses various of the aforementioned aspects. Specifically, this Research Topic’s cross-cutting theme is on collective movement, with papers investigating areas of pattern formation through formal methods and models, error handling, and dynamic exploration.

## 2 Content of the Research Topic

Giusti et al. address the problem of geometric pattern formation in large-scale robotic systems. Most specifically, the authors propose a distributed geometric approach allowing groups of robots to displace in triangular and square lattices, without communication and with limited sensing requirements. The approach is verified in simulation for robustness as well as in real settings using Robotarium. This works shows once again the significance of collective movement and the spatial dimension in collective systems, the need for *decentralised* and *low-resource* algorithms for driving collective behaviour, the method of numerical and experimental validation by simulation as an alternative for analytical verification, and the modern research practice of making artifacts available for reproducibility.^1^

The foundation for the research in Kano et al. is to reduce the workload of transportation industry personnel by introducing automated transport robots inside warehouses. The robots must move throughout the warehouse efficiently, with low energy consumption and collision avoidance. The authors reference numerous works in multi-robot/multi-agent systems, collision prediction and avoidance, and addressing multi-objective tasks. Their claim is that these efforts, including their own prior work, do not combine speed, smooth and safe movement, and collision avoidance with a minimalist approach to the environmental sensing needed for movement prediction of the robots in this domain. In this paper, the authors introduce a decentralized control scheme that relies on active sensing, which distinguishes between important and useless sensing information. The authors focus active sensing on agents that are expected to collide in the near future given a designated environmental view. Each agent independently predicts the future motion of agents within its view with a high collision risk and determines how to avoid them. The authors test their approach under different parameters, such as the number of agents in the environment, the size and angle of their view, and the threshold of collision risk. Overall, the new model performs well, while balancing the tradeoff between reduced sensing and calculation costs and potential collision avoidance.

Baumann et al. investigate *microscopic models of flocking*, together with the implications of their choice. The evaluation is performed using an accurate *submicroscopic* simulator, to measure the faithfulness of the studied models with respect to high-fidelity simulations of reality. Three microscopic models, based on different assumptions and modelling techniques, are analysed, showing that each one leads to different trade-offs between cost, and modelling, calibration, and implementation effort. Future work is needed to validate the results for more general settings.

Khenifar et al. introduce Collective Product Exploration (COPE): an approach by which outputs from one MAS can be consumed by another when two such systems are independently designed. This is an increasingly important challenge as the diversity and number of cyber-physical collectives grow. Unlike many works that would phrase the collaboration of different systems as a single heterogeneous multi-agent system, this work proposes to consider them as coupling two or more complex systems. In doing so, different considerations of physical and non-physical considerations are made. This is enabled through *meta-agents*, which are responsible for sensing and manipulating the information perceived by agents of the respective MAS to promote or incentivise certain behaviours. Significant description of a proposed COPE meta-architecture, message format, sequence diagrams are provided, as well as a motivating use case of wireless sensor network fire detection system coupled to a UAV is presented to motivate the work, as well as a simulation study to demonstrate the approach via network traffic analysis. The genericity of the approached is called out, as well as future desires to explore the scalability of their approach. The latter point being essential given the growth of cyber-physical collectives.

Buchanan et al. investigate the impact of diverse error models in multi-robot systems where each robot is governed by an autonomous software agent. Error diversity, where robots have their personal error model, is in contrast to error uniformity, where all robots or agents have the exact same error model, independent of the individual state and experience. They show in simulation and real-world experiments, that a uniform error model, fitted to all robots in a swarm, does not necessarily result in the desired swarm behaviour—even when the physical robots are presumably identical. Furthermore, they show that heterogeneous errors may include robots with high-error rates, harming the performance of the collective task. By identifying and replacing these robots, higher collective performances can be achieved.

Aldini provides a general-purpose process algebraic framework for the specification and analysis of collective adaptive systems. The author shows how the modelling approach can be instantiated to peculiar domains like trustworthy networks (Aldini, 2018) and peer-to-peer blockchain networks, and accordingly extended to account for quantitative analysis and, e.g., stochastic behaviour. This work is positioned in the long-standing tradition of formal modelling approaches and calculi for concurrent and distributed systems (Baeten and Reniers, 2010; Wirsing et al., 2023), and coordination languages (Ciatto et al., 2020), with peculiar extensions to model interaction (e.g., neighbour-based and community-based) and topologies of collective systems, and to favour syntactical composition of large networks of agents. The main benefit of the proposed framework is its abstraction and flexibility, resulting by the combination of an action-based formalism with data-driven and group-based communication mechanisms (cf. Alrahman et al., 2020) and the possibility of semantics customisation.

Kwa et al. provide a review and classification of approaches to *balance collective exploration and exploitation* in multi-agent systems situated in dynamic environments. The authors broadly classify the existing research contributions on the theme as either an *Agent Response Method* (based on changing agent behaviour in response to local environment changes or novel information from neighbours) or an *Information Dissemination Method* (based on adapting how information flows around the system, by direct or indirect/stigmergic methods). The review finds out, among other things, that metrics and strategies tend to be scenario-specific, with limited flexibility to different task and environments, and that adopting “stress benchmarks” for adaptivity is key but also challenging (e.g., due to the difficulty of capturing all the factors affecting actual results in real systems)—cf. Francesca and Birattari (2016).

## 3 Conclusion

This Research Topics includes contributions related to *understanding and engineering cyber-physical collectives*. In particular, the review on balancing collective exploration and exploitation by Kwa et al. provides insights applicable to different multi-agent domains. The approach of Khenifar et al. promotes the integration of the “collective product” of CPCs with other systems, e.g., to influence agents (cf. Casadei et al., 2021). Aldini shows the path of formal languages to support the flexible modelling and verification of CPCs (cf. Wirsing et al., 2023). The mobility of agents in CPCs is addressed by Giusti et al., covering geometric pattern formation, Baumann et al., proposing microscopic models for collective movement such as flocking, and Kano et al., proposing an active sensing-based control strategy for efficient collision avoidance. Another interesting research is carried out by Buchanan et al., who study the impact of different *error models* on foraging task partitioning—an aspect that would deserve a general investigation across various domains and tasks.

Though addressing different aspects of CPC engineering, these proposals generally rely on *simulations* to evaluate their effectiveness—showing once again the methodological importance of simulation but also the variety of simulators and the limited possibility of real-world experimentation. Indeed, simulation can focus on different aspects and properties of the system at hand, to achieve different trade-offs in research design. However, it is worth noticing that verification is not limited to simulation, and that, in several cases, formal specifications pave the way for model checking (cf. Aldini) or correct-by-construction techniques (cf. Bozga and Sifakis, 2023).

Another aspect that emerges is the variety of methodologies and approaches in use, ranging across formal, algorithmic, and more practical, engineering-oriented solutions. Despite difficulties and differences, the research clearly shows the importance of considering a *collective* dimension in the engineering of socio-technical systems (Abowd, 2016; Casadei, 2023a; Galesic et al., 2023), as a key complement to the traditional viewpoints and especially to the classical workflow of individually-engineered entities integrated together as a second step. Indeed, considering sets of devices as collectives enables the use of qualitatively different tools, e.g., macro-level abstractions and models. These help to hide low-level concerns, which can then be resolved and optimised by the platform or middleware, and to promote the specification of collaborative behaviour.

Last but not least, we observe that there is variability in the scope of contributions. For instance, while certain works address specific problems (e.g., pattern formation) under peculiar assumptions (e.g., limited sensing), others propose general solutions (e.g., architectures and frameworks) that can be tailored to multiple kinds of scenarios. As pointed out in Section 1, the field is quite fragmented around problems, methods, and domains. Generally speaking, increasing the understanding of what it means for a system to be a collective, and to exhibit collective intelligence, may promote the identification of rather *universal* laws and techniques for analysing and engineering collective systems in general.

## Footnotes

1
https://github.com/diBernardoGroup/SwarmSimPublic/tree/SwarmSimV1

## References

[B1] Abeywickrama D. B. Bicocchi N. Mamei M. Zambonelli F. (2020). The SOTA approach to engineering collective adaptive systems. Int. J. Softw. Tools Technol. Transf. 22, 399–415. 10.1007/S10009-020-00554-3

[B2] Abowd G. D. (2016). Beyond weiser: from ubiquitous to collective computing. Computer 49, 17–23. 10.1109/MC.2016.22

[B3] Aguilar W. Santamaría-Bonfil G. Froese T. Gershenson C. (2014). The past, present, and future of artificial life. Front. Robotics AI 1. 10.3389/frobt.2014.00008

[B4] Aguzzi G. Casadei R. Viroli M. (2022). “Towards reinforcement learning-based aggregate computing,” in Coordination Models and Languages - 24th IFIP WG 6.1 International Conference, COORDINATION 2022, Proceedings (Lucca, Italy: Springer), 72–91.

[B5] Aguzzi G. Casadei R. Viroli M. (2023). “Macroswarm: a field-based compositional framework for swarm programming,” in Coordination Models and Languages - 25th IFIP WG 6.1 International Conference, COORDINATION 2023, Proceedings (Lisbon, Portugal: Springer), 31–51.

[B6] Aldini A. (2018). Design and verification of trusted collective adaptive systems. ACM Trans. Model. Comput. Simul. 28, 9:1–9. 10.1145/3155337

[B7] Alrahman Y. A. Nicola R. D. Loreti M. (2020). Programming interactions in collective adaptive systems by relying on attribute-based communication. Sci. Comput. Program. 192, 102428. 10.1016/J.SCICO.2020.102428

[B8] Annaswamy A. M. Khargonekar P. P. Lamnabhi-Lagarrigue F. Spurgeon S. K. (2023). Cyber–physical–human systems: fundamentals and applications (Wiley). 10.1002/9781119857433

[B9] Baeten J. C. Reniers M. A. (2010) Process algebra: equational theories of communicating processes. Cambridge, United Kingdom: Cambridge University Press, 50.

[B10] Bellman K. L. Botev J. Diaconescu A. Esterle L. Gruhl C. Landauer C. (2021). Self-improving system integration: mastering continuous change. Future Gener. comput. Syst. 117, 29–46. 10.1016/J.FUTURE.2020.11.019

[B11] Boissier O. Bordini R. H. Hubner J. Ricci A. (2020) Multi-agent oriented programming: programming multi-agent systems using JaCaMo. Cambridge, MA: Mit Press.

[B12] Bozga M. Sifakis J. (2023). Correct by design coordination of autonomous driving systems. Int. J. Softw. Tools Technol. Transf. 25, 625–639. 10.1007/s10009-023-00723-0

[B13] Brambilla M. Ferrante E. Birattari M. Dorigo M. (2013). Swarm robotics: a review from the swarm engineering perspective. Swarm Intell. 7, 1–41. 10.1007/S11721-012-0075-2

[B14] Bucchiarone A. Mongiello M. (2019). “Ten years of self-adaptive systems: from dynamic ensembles to collective adaptive systems,” in From software engineering to formal methods and tools, and back - essays dedicated to stefania gnesi on the occasion of her 65th birthday. Editors ter Beek M. H. Fantechi A. Semini L. (Springer), 19–39. 10.1007/978-3-030-30985-5_3

[B15] Buchberger B. (2023). Automated programming, symbolic computation, machine learning: my personal view. Ann. Math. Artif. Intell. 91, 569–589. 10.1007/s10472-023-09894-7

[B16] Casadei R. (2023a). Artificial collective intelligence engineering: a survey of concepts and perspectives. Artif. Life 29, 433–467. 10.1162/ARTL_A_00408 37432100

[B17] Casadei R. (2023b). Macroprogramming: concepts, state of the art, and opportunities of macroscopic behaviour modelling. ACM Comput. Surv. 55, 275:1–37. 10.1145/3579353

[B18] Casadei R. Fortino G. Pianini D. Placuzzi A. Savaglio C. Viroli M. (2022). A methodology and simulation-based toolchain for estimating deployment performance of smart collective services at the edge. IEEE Internet Things J. 9, 20136–20148. 10.1109/JIOT.2022.3172470

[B19] Casadei R. Pianini D. Viroli M. Weyns D. (2021). Digital twins, virtual devices, and augmentations for self-organising cyber-physical collectives. Appl. Sci. 12, 349. 10.3390/app12010349

[B20] Cassandras C. G. Lafortune S. (2008) Introduction to discrete event systems. Springer.

[B21] Ciatto G. Mariani S. Serugendo G. D. M. Louvel M. Omicini A. Zambonelli F. (2020). Twenty years of coordination technologies: COORDINATION contribution to the state of art. J. Log. Algebr. Methods Program 113, 100531. 10.1016/J.JLAMP.2020.100531

[B22] Csuhaj-Varjú E. Dassow J. Kelemen J. Páun G. (2018) Grammar systems: a grammatical approach to distribution and cooperation. London, United Kingdom: Routledge. 10.4324/9781315075617

[B23] de Lemos R. Giese H. Müller H. A. Shaw M. Andersson J. Litoiu M. (2010). “Software engineering for self-adaptive systems: a second research roadmap,” in Software engineering for self-adaptive systems II - international seminar (Springer), 1–32. 10.1007/978-3-642-35813-5_1

[B24] Dorigo M. Theraulaz G. Trianni V. (2021). Swarm robotics: past, present, and future. Proc. IEEE 109, 1152–1165. 10.1109/JPROC.2021.3072740

[B25] Fernandez-Marquez J. L. Serugendo G. D. M. Montagna S. Viroli M. Arcos J. L. (2013). Description and composition of bio-inspired design patterns: a complete overview. Nat. Comput. 12, 43–67. 10.1007/S11047-012-9324-Y

[B26] Francesca G. Birattari M. (2016). Automatic design of robot swarms: achievements and challenges. Front. Robotics AI 3. 10.3389/frobt.2016.00029 PMC7806002 33501074

[B27] Galesic M. Barkoczi D. Berdahl A. M. Biro D. Carbone G. Giannoccaro I. (2023). Beyond collective intelligence: collective adaptation. J. R. Soc. Interface 20. 10.1098/rsif.2022.0736 PMC10031425 36946092

[B28] Gershenson C. (2023). Emergence in artificial life. Artif. Life 29, 153–167. 10.1162/ARTL_A_00397 36787448

[B29] Harvey P. Tatar A. Imai P. Wong L. Bringuier L. (2021). Evolutionary autonomous networks. J. ICT Stand. 9, 201–228. 10.13052/jicts2245-800X.927

[B30] Hendler J. Berners-Lee T. (2010). From the semantic web to social machines: a research challenge for AI on the world wide web. Artif. Intell. 174, 156–161. 10.1016/J.ARTINT.2009.11.010

[B31] Horling B. Lesser V. R. (2004). A survey of multi-agent organizational paradigms. Knowl. Eng. Rev. 19, 281–316. 10.1017/S0269888905000317

[B32] Júnior I. G. S. Santana T. S. Bulcão-Neto R. F. Porter B. (2022). The state of the art of macroprogramming in IoT: an update. J. Internet Serv. Appl. 13, 54–65. 10.5753/JISA.2022.2372

[B33] Kephart J. O. Chess D. M. (2003). The vision of autonomic computing. Computer 36, 41–50. 10.1109/MC.2003.1160055

[B34] Kim K. Kumar P. R. (2012). Cyber-physical systems: a perspective at the centennial. Proc. IEEE 100, 1287–1308. 10.1109/JPROC.2012.2189792

[B35] Klavins E. Ghrist R. Lipsky D. (2006). A grammatical approach to self-organizing robotic systems. IEEE Trans. Autom. Control 51, 949–962. 10.1109/TAC.2006.876950

[B36] Malone T. Bernstein M. (2022) Handbook of collective intelligence. MIT Press.

[B37] Malone T. W. Crowston K. (1994). The interdisciplinary study of coordination. ACM Comput. Surv. 26, 87–119. 10.1145/174666.174668

[B38] Mascardi V. Weyns D. Ricci A. (2019). Engineering multi-agent systems: state of affairs and the road ahead. ACM SIGSOFT Softw. Eng. Notes 44, 18–28. 10.1145/3310013.3310035

[B39] Pianini D. Casadei R. Viroli M. Natali A. (2021). Partitioned integration and coordination via the self-organising coordination regions pattern. Future Gener. comput. Syst. 114, 44–68. 10.1016/J.FUTURE.2020.07.032

[B40] Pinciroli C. Beltrame G. (2016). Buzz: a programming language for robot swarms. IEEE Softw. 33, 97–100. 10.1109/MS.2016.95

[B41] Roli A. Braccini M. (2018). Attractor landscape: a bridge between robotics and synthetic biology. Complex Syst. 27, 229–248. 10.25088/complexsystems.27.3.229

[B42] Stolfi D. H. Alba E. (2018). Green swarm: greener routes with bio-inspired techniques. Appl. Soft Comput. 71, 952–963. 10.1016/J.ASOC.2018.07.032

[B43] Viroli M. Beal J. Damiani F. Audrito G. Casadei R. Pianini D. (2019). From distributed coordination to field calculus and aggregate computing. J. Log. Algebr. Methods Program 109. 10.1016/J.JLAMP.2019.100486

[B44] Wirsing M. Jähnichen S. Nicola R. D. (2023). Rigorous engineering of collective adaptive systems - 2nd special section. Int. J. Softw. Tools Technol. Transf. 25, 617–624. 10.1007/S10009-023-00734-X

[B45] Wooldridge M. J. (2009) An introduction to MultiAgent systems. Second Edition. Wiley.

[B46] Zhang K. Yang Z. Basar T. (2019) Multi-agent reinforcement learning: a selective overview of theories and algorithms. Handbook of reinforcement learning and control, 321–384.

